# Supplementary material for: SyNDI: synchronous network data integration framework
Source: BMC Bioinformatics. 2018 Nov 6;19:403. doi: 10.1186/s12859-018-2426-5 (PMC6219086; doi:10.1186/s12859-018-2426-5)
Supplement: Supplementary file 5 — Files of the example presented in Additional file 4. This additional file is a zip package that contains all files used in Additional file 4. Detailed descriptions of each of these files are presented in a README file included in this zip package. (GZ 30411 kb) [file 12859_2018_2426_MOESM5_ESM.gz › SyNDITools/Meme2Fimo/meme_4.9.0_4.tar.gz!/meme_4.9.0/doc/qvalue.xml]

qvalue


# qvalue

**Usage:** `qvalue [options] <pvalues>`

**Description:**

Takes as input a list of p-values and prints a corresponding list of
q-values, computed using the method of Benjamini and Hochberg
(*Journal of the Royal Statistical Society B*, 57:289-300, 1995).

**Input:**

The input file contains one p-value per line. The p-values do not
have to be in sorted order.

**Output:**

The output is two tab-delimited columns, p-value and q-value, sorted
by p-value.

**Options:**


**Warning messages:** None

**Bugs:**

None known.
